# The Magnesium Status and Suggested Reference Ranges of Plasma Magnesium, Calcium, and Calcium/Magnesium Ratio in Chinese Adults over 45 Years Old

**DOI:** 10.3390/nu15040886

**Published:** 2023-02-09

**Authors:** Jingxin Yang, Yang Cao, Xiaoyun Shan, Huidi Zhang, Jie Feng, Jiaxi Lu, Lichen Yang

**Affiliations:** National Institute for Nutrition and Health, Chinese Center for Disease Control and Prevention, Key Laboratory of Trace Element Nutrition, National Health Commission of the People’s Republic of China, Beijing 100050, China

**Keywords:** magnesium, calcium, Ca/Mg ratio, reference range, Chinese adults

## Abstract

Magnesium (Mg) is an essential nutrient that participates in various enzymatic reactions and regulates important biological functions. The distribution and reference ranges in China have not been reported in populations more than 45 years old. This study aimed to assess the magnesium status and determine the reference values of plasma Mg, Ca, and Ca/Mg ratios for China’s population more than 45 years old. A total of 2101 people were randomly selected from the China Nutrition and Health surveillance (CNHS) (2015–2017), considering the regional types and monitoring points. Then, 337 healthy individuals were further selected by a series of strict inclusion criteria to explore the reference range. The plasma magnesium and calcium were tested by inductively coupled plasma mass spectrometry (ICP-MS). The suggested reference values for plasma Mg, Ca, and Ca/Mg ratios were 0.75–1.14 mmol/L, 2.17–3.64 mmol/L, and 2.36–3.66, respectively. Taking 0.75 mmol/L as the lower cut-off limit, the prevalence of Mg deficiency was 6.66%, and the average level of plasma magnesium was 0.88 mmol/L for populations older than 45 years in China. In conclusion, this study provides the magnesium status and reference ranges for plasma Mg, Ca, and Ca/Mg ratio for Chinese people over 45 years old. The results of the recommended reference ranges in this study were very similar to our published results in women of reproductive age. Thus, the reference range of plasma magnesium in different populations in China was further improved.

## 1. Introduction

Magnesium (Mg) is an essential nutrient that participates in various enzymatic reactions and regulates important biological functions [1]. Chronic Mg deficiency is associated with an increased risk of many clinical outcomes, including atherosclerosis [2], hypertension [3], altered lipid metabolism, insulin resistance [4], metabolic syndrome [5], cardiac arrhythmias, and stroke [6]. In addition, Mg deficiency may be at least a pathophysiological link that may help explain the interaction of inflammation and oxidative stress with the aging process and many age-related diseases [7]. However, there are still some problems in the nutritional status evaluation of Mg. Therefore, there are very limited reports on Mg nutrition in our population. On the one hand, although Mg deficiency can have serious health consequences, it has received little attention in clinical practice. This may be because, as compared with other elements such as sodium and potassium, there is no uniform, evidence-based reference range for plasma Mg [8]. Even so, plasma Mg is still the most commonly used indicator of Mg’s nutritional status [9]. The prevalence of Mg deficiency in people over 45 years old also has not been reported in China.

On the other hand, an imbalance of plasma Ca and Mg, especially high Ca levels, is closely associated with a variety of diseases [10]. Although the body’s Ca/Mg ratio is associated with a variety of diseases, there is still no consensus on the body’s appropriate Ca/Mg ratio. DAI-QI [11] conducted a case–control study to explore the optimal Ca/Mg ratio, and the results showed that high-grade prostate cancer was significantly associated with low blood Mg levels and high blood Ca/Mg ratios. Pamela Lutsey [12] used the data of a large population-based prospective cohort (ARIC) and certified that serum Mg was inversely associated with the development of heart failure (HF), and high serum phosphorus or Ca was independently associated with a greater risk of incident HF. Diet is the main source of nutrients; there is a traditional recommendation to keep the Ca/Mg ratio in the diet close to 2.0 for optimal health outcomes, but this recommendation comes from knowledgeable speculation and has not been approved by any experimental evidence [13]. Our team has published blood reference ranges [14] for Mg, Ca, and Ca/Mg ratio in women of childbearing age 18–45 years: 0.75–1.13 mmol/L, 2.27–3.43 mmol/L, and 2.41–3.44, respectively. This study included different ages and genders to investigate whether these reference ranges are different. Therefore, the purpose of this study was to assess the magnesium status and establish reference values of plasma Mg, Ca, and Ca/Mg ratio in adults in the population aged ≥ 45 years old in representative national surveillance.

## 2. Materials and Methods

### 2.1. Study Subjects

This study’s data were based on the China Nutrition and Health Surveillance (2015–2017) (CNHS), a nationally representative cross-sectional survey. In each of the 302 monitoring sites in China, multi-stage cluster random sampling was used to select the survey objects. Detailed sampling strategies are available in the previous article [15]. The sample size-calculating formula of the cross-sectional study was N = $deff\frac{u^{2}p(1-p)}{d^{2}}$. According to Limin Wang’s research, the prevalence of diabetes in Chinese in 2013 was 10.9% [16]. The values of the parameters were a *u* of 1.96, a *p* of 0.109, a *deff* of 1.5, and a *d* of 4%. Considering the regional types and monitoring sites, the minimum sample size was 2100 people. Finally, a total of 2101 participants were included in this study. Then, 337 individuals were further selected using a series of strict inclusion criteria to explore the reference range. The inclusion criteria included the following indicators: Body Mass Index (BMI, 18.5–24.0 kg/m^2^); blood pressure (SBP, 90–140 mmHg and DBP, 60–89 mmHg); blood glucose (fasting plasma glucose, 3.9–5.59 mmol/L and hemoglobin, 4–5.69%); blood lipids (TC, <5.2 mmol/L and TG, 0.56–1.7 mmol/L and LDL-C, <3.12 mmol/L and HDL-C, 1.04–2.07 mmol/L); uric acid (UA, ≤360 µmol/L); hemoglobin (Hb, 115–150 g/L) and heart rate (60–100 t/min). This study has been approved by the Ethics Committee of the National Institute of Nutritional and Health, and the Chinese Center for Disease Control and Prevention (CDC) (Number: 201519-A). The study was also in compliance with the Declaration of Helsinki. All subjects signed written informed consent.

### 2.2. Basic Information Collection

The study collected the basic information through a standardized questionnaire. The participants’ venous blood was divided into different tubes after being centrifuged at 3000× *g* for 15 min. Then, the blood was frozen at −80 °C for subsequent analysis. Their smoke status, drink status, nationality, and education experience were recorded on the questionnaire. According to the type of city, urban and rural residences were divided. According to their location in China, they were divided into three areas: east, central, and west. Those who attended elementary school or below, middle school or high school, and college or above were defined as a low, medium, or high level of education, respectively. The trained staff examined the participants’ weight, height, waist, and blood pressure. BMI was calculated by dividing weight (kg) by squared height (m).

### 2.3. Laboratory Index Detection

The study measured enzymatically indexes including blood glucose, lipid, and uric acid by an automatic biochemical analyzer (Hitachi 7600, Tokyo, Japan). We used the high-performance lipid chromatography (HPLC) method in Trinity Biotech, Premier Hb9210 (Dublin, Ireland) to measure HbA1c. The concentrations of Mg and Ca in plasma were measured by Inductively Coupled Plasma Mass Spectrometry (ICP-MS, PerkinElmer, NexION 350, Waltham, MA, USA). The test method was accredited by the International Federation of Clinical Chemistry (IFCC). All laboratories participating in this surveillance have passed the External Quality Assessment National Clinical Laboratory Center.

The study used 0.5% (*v*/*v*) high-purity nitric acid and diluted the sample to a rate of 1:20. This study also used the market quality control samples (Seronorm, Level 2, Billingstad, Norway; Clinchek Level 2, Munich, Germany) in 20-sample intervals to monitor the precision and accuracy of the analysis. The coefficient of variation between and within batches of Mg was 3.56% and 2.30%, respectively. The coefficient of variation of Ca between and within batches was 1.36% and 2.31%, respectively.

### 2.4. Statistical Analysis

SAS version 9.4 software was used for all statistical data cleaning and analysis. The results for descriptive characteristics were expressed as geometric mean (Mean), median (P50), P2.5, P25, P75, and P97.5. The International Union of Clinical Chemistry (IFCC) recommends at least 120 observations to estimate reliable reference values [17]. They recommend that 2.5% and 97.5% of clinical biomarkers define the reference intervals in the reference population. The *t*-test was used to test the differences between the two groups, and the one-way ANOVA was used to detect the difference between more than two groups. The comparison of percentages used the chi-square test. All *p*-values were bilateral, and *p*-values < 0.05 were considered statistically significant differences.

## 3. Results

### 3.1. Basic Characteristics of 337 Healthy Participants

A total of 337 healthy individuals were included in this analysis after rigorous selection, and their basic information is summarized in Table 1. The mean age of the study population was 57.1 years old. Their BMI, waist, blood pressure, blood lipids, blood glucose, and UA were all within the normal range.

### 3.2. Plasma Concentrations of Mg, Ca, and Ca/Mg Ratio in Healthy Participants

The plasma concentrations of Mg, Ca, and Ca/Mg ratio of 337 healthy individuals were shown in Table 2. These values were stratified by gender, age, area, and residence. The reference ranges of Mg were 0.75–1.14 mmol/L, and there was no significant statistical difference under the stratification factors. The reference ranges of Ca were 2.17–3.64, and age had influence on it (*p* = 0.009). The Ca/Mg ratio ranges from 2.36 to 3.66. The Ca/Mg ratio differed among age and area subgroups. The group more than 60 years old had broader reference ranges than the 45–60-year-old group (*p* = 0.014). The central area also had broader reference ranges than the east and west areas *(p* = 0.016).

### 3.3. Plasma Mg Reference Ranges in Various Countries

Table 3 presents the reference ranges of plasma Mg in different age groups and the related measurement methods. The results of this study showed that the mean (0.91 mmol/L) and reference range of plasma Mg values (0.75–1.14 mmol/L) were similar to the published data by our team on Chinese women of reproductive age, and similar to the USA, Denmark, and Iran’s lower limit, while slightly higher than those in the UK and Sweden.

### 3.4. The Distribution and Deficiency of Plasma Magnesium in 2101 Participants

According to the lower limit of the reference range obtained above, the distribution and deficiency of plasma Mg in 2101 individuals are shown in Table 4. There was a significant difference in plasma Mg in the BMI sub-group. As BMI increased, plasma Mg showed a tendency to decrease. In different nationality and age sub-groups, plasma Mg also showed a decreasing trend, the *p*-value was close to 0.05. Plasma Mg did not differ significantly in different gender, area, residence, smoking status, drinking status, and education groups. Taking 0.75 mmol/L as the threshold value of Mg deficiency, the overall Mg deficiency rates were 6.66% (140/2101). The ethnic minority groups had a higher Mg deficiency rate than the Han group (9.83% vs. 6.27%, *p* = 0.039). The high education level group also had a higher Mg deficiency rate than the low and medium education group (9.38% vs. 7.85% and 4.76%, *p* = 0.018). No significant difference in the Mg deficiency rate was observed in different gender, age, area, residence, BMI, smoking status, and drinking status groups.

## 4. Discussion

Magnesium status is associated with the development of many diseases, and different ages and genders may have different Mg nutritional statuses or reference ranges [26]. Therefore, we used the latest nationally representative nutrition surveillance data to establish reference ranges for Mg, Ca, and Ca/Mg ratios. Some 337 healthy individuals were screened from 2101 participants aged more than 45 years old, according to strict criteria. To the best of our knowledge, this is the first description of plasma Mg nutritional status in a Chinese population over 45 years of age, and the first to use representative data from healthy individuals to establish a reference range for this age group.

The NHANES I (1971–1974) [18] was the first study that could be found that suggested a reference range for plasma Mg. Their results showed 95% of adults aged 18–74 years falling between 1.50 and 1.91 mEq/L (equal to 0.75–0.95 mmol/L). The lower limit of our study is exactly consistent with the 0.75 mmol/L of this study. Our upper limit of plasma Mg was higher than in this study. Except for the USA, our study’s lower limit of plasma Mg was also similar to Iran’s 20–50-year-old population [21]. Iran’s 3–19-year-old individuals [20] and Denmark’s 20–54-year-old healthy men [22] had a slightly higher standard than our results, close to 0.75 mmol/L. The UK [19] and Sweden’s [23,24,25] recommendation was lower than 0.75 mmol/L; this may be because the UK’s study controlled the calcium and alkaline phosphatase values, and these two countries used Xylidyl blue dye method to test plasma Mg, which is different with other countries. The ICP-MS method for plasma Mg has been developed [27]. This method was specific, precise, simple, and low in cost. P. Saur [28] used 30 patients’ plasma samples to compare the sensitivity of methylthymol blue spectrophotometry and AAS methods. The results showed that the methylthymol blue spectrophotometry method has higher results of the same plasma sample and low precision of a standardized plasma concentration. Therefore, the AAS and ICP-MS used in this study may have better accuracy and sensitivity for the detection of plasma Mg.

There was no significant difference between gender, age, regional distribution, and urban/rural area, which was similar to Lena Carlsson’s [23] cohort research. Carlsson investigated 1016 subjects to validate NORIP (Nordic Reference Interval Project) reference values in a 70-year-old population. Based on this cohort, the establishment of reference ranges at 75 [24] and 80 years [25] followed, and both certified that plasma Mg in different sexes and ages showed high concordance. Consistent with Carlsson’s cohort study, this study was conducted in a population older than 45 years, and previously published studies [14] were conducted in women of childbearing age, i.e, 18–45. Although the two studies differed in age and gender, the reference range for plasma Mg also showed high concordance. In this study, the reference range of Mg was 0.75–1.14 mmol/L. Zhang’s [14] reference range for plasma Mg was 0.75–1.13 mmol/L in Chinese women of childbearing age between 18–45 years. Both studies suggest that age may not be a factor in the reference range of plasma Mg in adults.

Recently, Oliver Micke [8] published an article titled ‘the Time for a Standardized and Evidence-Based Reference Range of Serum Mg’. They asserted that serum Mg values less than 0.85 mmol/L are associated with increased health risks. Unlike us, the cut-off value of 0.85 mmol/L for Micke’s study was developed based on clinical outcomes. Our study was performed with a traditional method, using the 2.5 and 97.5 percentile distributions of plasma Mg in a healthy population. A healthy person means all physical measurements and general biochemical tests were in the normal range and that the common chronic diseases, including hyperuricemia, hypertension, hyperlipidemia, and hyperglycemia were excluded.

The balance of Ca and Mg in the blood is vital to the body. This study’s Ca/Mg ratio reference range was 2.36–3.66. This ratio is also greater than that of 18–44-year-old Chinese childbearing women (2.41–3.44) [14]. Gatot Winarno’s research [29] used a receiver operating characteristic curve (ROC) analysis on the serum Ca/Mg ratio and showed that women with a serum Ca/Mg ratio of <2.36 were predicted to have a risk of preeclampsia. Qing Li [30] used the data of a prospective cohort study of 3380 coronary artery disease (CAD) patients to assess the associations of plasma Ca/Mg ratio with mortality in patients with CAD. Their result showed that those with a Ca/Mg ratio range of 3.91–4.70 had the lowest mortality risk, and that a low plasma Mg and high Ca/Mg ratio were independent risk factors of mortality in CAD patients. Rebecca Costello [31] found a rising trend in Ca/Mg ratio in the United States. To reduce the risk of multiple chronic diseases, individuals who consume large amounts of Ca from their diet may need to take Mg supplements to establish a more favorable Ca/Mg ratio in their total diet. Chenlu Yang [32] used the data of the Kailuan Cohort in China, including 1816 non-alcoholic fatty liver disease (NAFLD) cases and 1111 controls to explore the relationship between Ca/Mg ratio and NAFLD in Chinese adults. Their results showed that a lower Ca/Mg ratio was significantly associated with the risk of NAFLD. Although these studies have demonstrated an association between Ca/Mg ratios and disease, there are no other internationally agreed recommended reference ranges for Ca/Mg ratios.

Studies on the prevalence of Mg deficiency in the Chinese population are scarce and lack representative data. Zhang’s [14] research showed an Mg deficiency rate of 4.79% in women of childbearing age (18–45 years) according to a 0.75 cut-off value. The Mg deficiency rate in the current study (6.66%) was slightly higher than that study, possibly due to the wider age range of 45–91 years, and the deficiency rates of Mg may be higher in older age groups. However, when comparing the Mg deficiency rate by age group, although the prevalence rate showed an increasing trend with age, there was no statistical difference in this population. In addition to age, this study also found that plasma Mg levels decreased with increasing BMI. Similarly, Jennifer Morais [33] reviewed the related articles and showed that obese subjects have low serum concentrations of Mg, and found high concentrations of oxidative stress in obesity. Mohammadreza Askari [34]’s meta-analysis included 32 pieces of research to quantify the effect of Mg supplementation on obesity. The results showed that a great reduction in BMI was caused by Mg supplementation. Contrary to our results, Fernando Guerrero-Romero’s [35] cross-sectional study enrolled 681 healthy individuals to examine the relationship between body weight and Mg deficiency. Their results showed that BMI was not associated with Mg deficiency (i.e., a serum Mg concentration less than 0.74 mmol/L). Therefore, the relationship between BMI and plasma Mg needs more research to certify.

In this study, the reference range of plasma Ca was 2.17–3.64 mmol/L in total, which is wider than that of 18–44-year-old Chinese childbearing women (2.27–3.43 mmol/L) [14]. NORIP’s [36] suggested reference of plasma Ca was 2.17–2.51 mmol/L. Our upper limit is higher than NORIP, this may be due to race difference, or other confounding factors that need to be discovered. In the stratified comparison, the differences were statistically significant at 2.18–3.65 and 2.10–3.58 for those aged 45–60 and older than 60, respectively. As age increases, blood Ca levels show a significant decrease, which is consistent with older adults being more likely to suffer from osteoporosis and fractures [37]. Yin’s [38] study included 80 participants to explore the potential influencing factors of Ca metabolic balance. The results showed that age is important in calcium retention and absorption.

This study has several advantages. First, the subjects were from the CNHS 2015, which had a complete quality control system and was nationally representative. Second, the distribution of plasma Mg by age, sex, BMI, region, and rural/urban area was analyzed and compared in a stratified manner. Third, according to strict selection criteria, 337 healthy people over 45 years old were selected to explore the reference range; until now, there have been no relevant reports on this age group in China.

Nevertheless, this study also has several limitations. First, since the reference range research of plasma Ca and Ca/Mg ratios was scarce, a detailed comparison cannot be made. Second, although the study selected healthy individuals as representatives, it did not test their inflammatory markers or insulin status, and could not exclude the situation of inflammation or insulin resistance in the selected population. Third, the reference range established in this study was based on healthy participants and was not associated with diseases. The role of this reference range in diseases is unknown and needs further research.

## 5. Conclusions

In summary, we found the average level of Mg in people over 45 years old in China was 0.88 mmol/L, and the prevalence of Mg deficiency was 6.66%. This study also provides reference ranges for plasma Mg, Ca, and the Ca/Mg ratio for Chinese people over 45 years old. The study provides evidence for the Chinese population about the nutritional status of Mg. The results of the recommended reference ranges in this study were very similar to our published results in women of reproductive age. Thus, the reference range of plasma magnesium in different populations in China was further improved.

## Figures and Tables

**Table 1:** Basic characteristics of the 337 healthy participants.

Variables (N = 337)	Mean	Median	P25	P75
Age (years)	57.1	56.3	50.2	62.9
Height (cm)	159.60	159.10	154.00	165.40
Weight (kg)	54.86	54.70	50.30	59.30
BMI (kg/m^2^)	21.48	21.51	20.47	22.64
Waist (cm)	75.96	76.00	72.00	79.53
Blood lipids				
TC (mmol/L)	4.29	4.37	3.96	4.65
TG (mmol/L)	0.90	0.84	0.70	1.05
LDL-C (mmol/L)	2.44	2.50	2.14	2.76
HDL-C (mmol/L)	1.41	1.37	1.25	1.56
Blood pressure				
SBP (mmHg)	120	122	116	130
DBP (mmHg)	74	74	68	79
Blood glucose				
FPG (mmol/L)	4.90	4.96	4.73	5.20
HbA1c (%)	4.76	4.80	4.30	5.10
Others				
Hb (g/L)	146	147	136	158
UA (µmol/L)	261.05	259.00	214.50	306.50
Heart rate (t/min)	75	74	67	80

**Table 2:** Plasma concentrations of Mg, Ca, and Ca/Mg ratio in 337 healthy individuals (more than 45 years old).

Variables	N	Mg (mmol/L)					Ca (mmol/L)					Ca/Mg				
		Mean	Median	P2.5	P97.5	*p*	Mean	Median	P2.5	P97.5	*p*	Mean	Median	P2.5	P97.5	*p*
Total	337	0.91	0.90	0.75	1.14		2.65	2.53	2.17	3.64		2.90	2.84	2.36	3.66	
Gender						0.337					0.488					0.068
Male	161	0.92	0.90	0.76	1.15		2.63	2.53	2.16	3.62		2.86	2.80	2.30	3.64	
Female	176	0.91	0.89	0.75	1.14		2.66	2.53	2.17	3.65		2.93	2.87	2.40	3.74	
Age group						0.341					0.009					0.014
45-< 60 years	214	0.92	0.90	0.75	1.15		2.69	2.55	2.18	3.65		2.93	2.86	2.42	3.64	
60-	123	0.91	0.88	0.74	1.13		2.56	2.49	2.10	3.58		2.84	2.78	2.25	3.69	
Area						0.988					0.099					0.016
East	109	0.91	0.90	0.75	1.11		2.59	2.50	2.19	3.62		2.84	2.81	2.29	3.54	
Mid	104	0.91	0.89	0.71	1.16		2.71	2.55	2.08	3.67		2.97	2.86	2.41	3.72	
West	124	0.91	0.90	0.76	1.15		2.64	2.50	2.21	3.63		2.90	2.84	2.37	3.74	
Residences						0.176					0.157					0.597
City	169	0.91	0.89	0.75	1.14		2.61	2.51	2.12	3.63		2.89	2.85	2.37	3.61	
Rural area	168	0.92	0.91	0.75	1.14		2.68	2.54	2.19	3.64		2.91	2.83	2.31	3.73	

**Table 3:** The reference ranges of plasma Magnesium in different countries.

Regions	N	Participants	Method	Mean Plasma Mg (mmol/L)	Reference Range of Plasma Mg (mmol/L)	Published Year
China (This study)	337	45-year-old healthy individuals	ICP-MS ^1^	0.91	0.75–1.14	-
China [14]	182	18–44-year-old healthy women	ICP-MS ^1^	0.90	0.75–1.13	2021
USA [18]	5191	18–74-year-old individuals	AAS ^2^	0.85	0.75–0.96	1986
UK [19]	800	20-year-old individuals	Xylidyl blue dye	0.81	0.64–1.03	1990
Iran [20]	306	3–19-year-old individuals	AAS ^2^	0.86	0.76–0.99	2010
Iran [21]	491	20–50-year-old individuals	AAS ^2^	0.87	0.75–1.05	2010
Denmark [22]	60	20–54-year-old healthy men	AAS ^2^	0.87	0.765–0.997	1995
Uppsala, Sweden [23]	1016	70-year-old individuals	Xylidyl blue dye	NR ^3^	Male: 0.71–0.96/Female 0.71–0.96	2010
Uppsala, Sweden [24]	727	75-year-old population	Xylidyl blue dye	NR ^3^	Male: 0.72–0.94/Female 0.70–0.93	2012
Uppsala, Sweden [25]	531	80-year-old population	Xylidyl blue dye	NR ^3^	Male: 0.71–1.12/Female 0.68–1.11	2016

^1^ ICP-MS, inductively coupled plasma mass spectrometry. ^2^ Atomic absorption spectrometry. ^3^ Not reported.

**Table 4:** Plasma magnesium concentrations and deficiency in the 2101 participants.

Variables	N	Mg (mmol/L)					Mg Deficiency (%)	*p*
		Mean	Median	P25	P75	*p*		
Total	2101	0.88	0.87	0.82	0.93		6.66%	
Gender						0.740		0.354
Male	1046	0.88	0.87	0.82	0.93		7.17%	
Female	1055	0.88	0.87	0.82	0.93		6.16%	
Nationality						0.067		0.039
Han	1867	0.88	0.87	0.82	0.93		6.27%	
Ethnic minorities	234	0.87	0.86	0.81	0.92		9.83%	
Age group						0.051		0.547
45 < age ≤ 55	548	0.89	0.88	0.82	0.94		5.47%	
55 < age ≤ 65	701	0.88	0.87	0.81	0.93		6.70%	
65 < age ≤ 75	411	0.88	0.88	0.82	0.93		7.06%	
Age > 75	441	0.88	0.88	0.82	0.94		7.71%	
Area						0.327		0.373
East	744	0.88	0.88	0.82	0.94		5.78%	
Mid	625	0.88	0.87	0.82	0.94		7.68%	
West	732	0.88	0.87	0.81	0.93		6.69%	
Residences						0.149		0.086
City	1255	0.88	0.88	0.82	0.94		5.90%	
Rural area	846	0.88	0.87	0.82	0.93		7.80%	
BMI group						0.002		0.236
<18.5	86	0.89	0.89	0.83	0.95		9.30%	
18.5–23.9	1001	0.89	0.88	0.82	0.94		7.09%	
24–27.9	707	0.88	0.87	0.82	0.92		5.23%	
≥28	307	0.86	0.86	0.81	0.91		7.82%	
Smoke						0.330		0.197
Yes	534	0.88	0.87	0.81	0.93		7.87%	
No	1567	0.88	0.88	0.82	0.93		6.25%	
Drink						0.845		0.725
Yes	692	0.88	0.88	0.82	0.93		6.94%	
No	1409	0.88	0.87	0.82	0.93		6.53%	
Education						0.103		0.018
Low	1249	0.88	0.87	0.81	0.93		7.85%	
Medium	820	0.89	0.88	0.83	0.94		4.76%	
High	32	0.87	0.86	0.83	0.92		9.38%	

## Data Availability

Not applicable.
